# Alcohol Promotes Exosome Biogenesis and Release *via* Modulating Rabs and miR-192 Expression in Human Hepatocytes

**DOI:** 10.3389/fcell.2021.787356

**Published:** 2022-01-14

**Authors:** Shashi Bala, Mrigya Babuta, Donna Catalano, Aman Saiju, Gyongyi Szabo

**Affiliations:** ^1^ Department of Medicine, Beth Israel Deaconess Medical Center, Harvard Medical School, Boston, MA, United States; ^2^ Department of Medicine, University of Massachusetts Medical School, Worcester, MA, United States

**Keywords:** alcohol, exosomes, rabs, syntenin, syntaxin, VAMPs, miR-192, miR-122

## Abstract

Exosomes are membrane vesicles released by various cell types into the extracellular space under different conditions including alcohol exposure. Exosomes are involved in intercellular communication and as mediators of various diseases. Alcohol use causes oxidative stress that promotes exosome secretion. Here, we elucidated the effects of alcohol on exosome biogenesis and secretion using human hepatocytes. We found that alcohol treatment induces the expression of genes involved in various steps of exosome formation. Expression of Rab proteins such as Rab1a, Rab5c, Rab6, Rab10, Rab11, Rab27a and Rab35 were increased at the mRNA level in primary human hepatocytes after alcohol treatment. Rab5, Rab6 and Rab11 showed significant induction in the livers of patients with alcohol-associated liver disease. Further, alcohol treatment also led to the induction of syntenin, vesicle-associated membrane proteins (VAMPs), and syntaxin that all play various roles in exosome biogenesis and secretion. VAMP3, VAMP5, VAPb, and syntaxin16 mRNA transcripts were increased in alcohol treated cells and in the livers of alcohol-associated liver disease (ALD) patients. Induction in these genes was associated with increases in exosome secretion in alcohol treated hepatocytes. We found that hepatocyte enriched miR-192 and miR-122 levels were significantly decreased in alcohol treated hepatocytes whereas their levels were increased in the cell-free supernatant. The primary transcripts of miR-192 and miR-122 were reduced in alcohol treated hepatocytes, suggesting alcohol partially affects these miRNAs at the transcriptional level. We found that miR-192 has putative binding sites for genes involved in exosome secretion. Inhibition of miR-192 in human hepatoma cells caused a significant increase in Rab27a, Rab35, syntaxin7 and syntaxin16 and a concurrent increase in exosome secretion, suggesting miR-192 regulates exosomes release in hepatocytes. Collectively, our results reveal that alcohol modulates Rabs, VAMPs and syntaxins directly and partly *via* miR-192 to induce exosome machinery and release.

## Introduction

Exosomes are small membrane extracellular vesicles (<100 nm or <200 nm) released by various cell types into the extracellular space under different physiological and pathological conditions (Trams et al., 1981; Bala et al., 2012; Bala et al., 2015; Thery et al., 2018)**.** Exosomes can be found in most biological fluids such as blood, saliva, and urine (Szabo and Momen-Heravi, 2017). Exosomes contain nucleic acids (for e.g., RNA and miRNA), proteins, lipids and other biomolecules that can modulate the function of the recipient cell. By transferring their cargos, exosomes emerged as a new mode of intercellular communication (Valadi et al., 2007; Momen-Heravi et al., 2015a). Exosome formation comprises of three steps: biogenesis, transport, and release. Briefly, exosomes are formed by inward invagination of the endosomal membrane the first step is the formation of intraluminal vesicles (ILVs) in multivesicular body (MVBs; late endosomes). MVBs are then transported to and fused with the plasma membrane resulting in the release of exosomes into the extracellular space (Hessvik and Llorente, 2018)**.** Various components, such as the endosomal sorting complexes required for transport (ESCRT) machinery, are involved in multivesicular endosome formation and syntenin is one of the proteins known to facilitates ILV formation (Baietti et al., 2012). Furthermore, syndecan-syntenin-ALIX are well recognized for their imperative role in exosomes biogenesis (Baietti et al., 2012).

The transport of MVBs to the plasma membrane is governed by various factors including small Rab GTPases. Till now, more than 70 Rab proteins has been identified. Through their association with the endocytic and secretion pathways, Rabs are widely known for their roles in membrane transport and fusion (Blanc and Vidal, 2018). Various Rabs such as Rab5a, Rab7, Rab11, Rab27a/b, and Rab35 are shown to require for exosome release (Savina et al., 2002; Hsu et al., 2010; Ostrowski et al., 2010; Blanc and Vidal, 2018; Hessvik and Llorente, 2018). Among all the Rab proteins, Rab11, Rab27 and Rab35 have been shown to play direct role in exosome biogenesis and secretion (Blanc and Vidal, 2018). Rab11 and Rab35 are reported to be involve in the recycling of membrane components from endosomal compartment to the plasma membrane (Hsu et al., 2010; Ramel et al., 2013). Whereas Rab27 was shown to involve in the transport of late endosomal/lysosome-like compartments to the plasma membrane (Ostrowski et al., 2010). Both isoforms of Rab27, Rab27a and Rab27b are involved in the exosome secretory pathway (Ostrowski et al., 2010).

Soluble N*-*ethylmaleimide-sensitive factor attachment protein receptors (SNAREs), proteins are involved in the final step of exosome secretion that is the fusion of MVBs to the plasma membrane (Jahn and Scheller, 2006). SNAREs are classified as either t-SNAREs (target membrane) and v-SNAREs (vesicle membrane). The vSNARE protein, VAMP7 was demonstrated to mediate the fusion of secretory lysosomes with the plasma membrane and to regulate of EVs release (Fader et al., 2009). Syntaxins belong to t-SNAREs proteins and play an important role in membrane trafficking and autophagy (Tang, 2019; Mori et al., 2021).

Liver resident cells including parenchyma (hepatocytes) and non-parenchymal cells are involved in the pathogenesis of alcohol-associated liver disease (ALD) (Osna et al., 2017). Alcohol is predominantly metabolized by hepatocytes that make up to 70 percent of the liver mass (Osna et al., 2017). Alcohol induces oxidative stress and previously we demonstrated an increase in exosome secretion in alcoholic hepatitis patients (Momen-Heravi et al., 2015b; Babuta et al., 2019), *in vivo* alcohol mouse models (Momen-Heravi et al., 2015a; Momen-Heravi et al., 2015b; Babuta et al., 2019) and *in vitro* model of alcohol-treated human hepatocytes (Momen-Heravi et al., 2015a). However, the effect of alcohol on genes involved in exosome biogenesis, transport and secretion is not known.

miRs are small noncoding RNAs that regulate gene expression and are involved in various physiological processes (Szabo and Bala, 2013; Babuta and Szabo, 2021). Altered miRNA expression has been found in ALD (Bala and Szabo, 2012; Bala et al., 2016; Satishchandran et al., 2018). Both miR-122 and miR-192 are highly expressed in hepatocytes (Momen-Heravi et al., 2015a) and a decrease in miR-122 levels was found in ALD (Bala et al., 2012) in Non-alcoholic fatty liver disease (NAFLD) (Csak et al., 2015) and a down-regulation in hepatic miR-192 levels was reported in NAFLD (Liu et al., 2017)**.** Since one miRNA can affect many target genes, thereby have a wider effect on multiple cellular pathways, we hypothesized that alcohol induces exosome secretion *via* affecting multiple factors including miRNA. Recently, we established a link between miR-155 and lysosome dysfunction in exosome release in ALD (Babuta et al., 2019). In this study, we demonstrated that alcohol induces the expression of Rabs, VAMPs, and syntaxins in primary human hepatocytes, human hepatoma cell line, Huh7.5 cells, and in ALD patients. Hepatocyte enriched miR-192 and miR-122 were found to be decreased and a significant increase in the number of exosomes was observed in primary human hepatocytes after alcohol treatment. Our mechanistic studies suggest that inhibition of miR-192 caused an increase in Rabs and syntaxins and a concurrent increase in exosome secretion in hepatocytes. Collectively, our work reveals following two important findings: first, alcohol affects multiple genes involved in exosome biosynthesis and release directly and partly *via* miR-192, and second, a novel role of miR-192 in exosome secretion in hepatocytes.

## Materials and Methods

### Primary Human Hepatocytes and Cell Lines

Primary human hepatocytes were obtained from the National Institutes of Health (NIH) liver tissue cell distribution system (LTCDS; Minneapolis, MN, United States; Pittsburgh, PA; Richmond, VA, United States), and were maintained in low-glucose without phenol red low-glucose Dulbecco’s modified Eagle medium (DMEM) media supplemented with 1% anti-anti (Gibco, Thermofisher Scientific, MA, United States), 1% Insulin transferrin (Gibco, Thermofisher Scientific, MA, United States) and 2% exosome depleted fetal bovine serum (System Bioscience, United States). For alcohol treatment, cells were treated with different dosages of alcohol (25 and 50 mM) and incubated in C.B.S. Scientific incubation culture chambers with twice the alcohol concentration in bottom of the chamber to saturate and maintain a stable alcohol concentration (25 mM or 50 mM) in the chamber.

Human hepatoma cell line, Huh7.5 were maintained and cultured in low-glucose DMEM containing 1% pencillin-streptomycin (Gibco, Thermofisher Scientific, MA, United States) and 10% exosome depleted fetal bovine serum (System Bioscience) and supplemented with nonessential amino acids (NEAA) (Gibco, Thermofisher Scientific, MA, United States). Cells were treated with 50 mM alcohol as described above for 6, 18, 36 and 48 h.

### Patient Samples

Human liver samples from control subjects, and patients with alcohol-associated liver disease (n = 8–10) were obtained from the National Institutes of Health Liver Tissue Cell Distribution System (Minneapolis, MN). The BIDMC Institutional Review Board for Protection of Human Subjects in Research approved the study. The criteria to define patients with ALD were based on “Recommendation from the NIAAA Alcoholic Hepatitis Consortia” (Crabb et al., 2016).

### RNA Extraction

Total RNA was extracted from the primary human hepatocytes or Huh7.5 cells or from the supernatant using Direct-zol RNA MiniPrep kit (Zymo Research, Irvine, CA) as described by the manufacturer. Synthetic Cel-miR-39 was spiked during the extraction of RNA from the supernatant and used as a normalization control as described previously (Momen-Heravi et al., 2015b). RNA was transcribed into cDNA with the iScript cDNA synthesis supermix kit (Bio-Rad Laboratories Inc, CA, United States). Real-time quantitative polymerase chain reaction was performed on CFX96 iCycler (Bio-Rad). For miRNA, TaqMan miRNA assays were used (Ambion, TX, United States) and RNU48 was used to normalize the data. Primary miR-122 and miR-192 levels were measured using TaqMan gene expression assay (Ambion, TX, United States) and GAPDH was used as a normalization control as described by the manufacturer. For mRNA quantification, 18s was used for normalization of cq values among the samples.

### NanoSight/Nanoparticle Tracking Analysis (NTA)

The concentration and size distribution of extracellular vesicles (EVs) from the cell culture supernatants were determined using the NanoSight NS300 system (NanoSight, Amesbury, United Kingdom) (Momen-Heravi et al., 2015a). The samples were captured for 30 s in triplicates at room temperature and NTA post-acquisition settings were kept constants for all samples. The NTA software was used to process the captured videos with setting as threshold of five arbitrary unit (AU) and screen gain of 5AU and concentration (particles/milliliter) and size distribution (in nanometers) were calculated from the measured particles. The particles ranging from 40 to 100 nm were defined as exosomes. In previous reports our laboratory demonstrated that this EV size corresponds to exosomes and express exosome markers including CD63 (Momen-Heravi et al., 2015a)

### Transfection

For miR-192 inhibition studies, Huh7.5 cells, human hepatocyte cell line, were either transfected with a negative control miRNA or miR-192 inhibitor at 30pM for 24 h (Applied Biosystems, Foster City, CA) using Lipofectamine RNAi max reagent (Thermo Fisher Scientific) as described by the manufacturer. Cells were harvested 48 h after transfection. Cell-free supernatants were used to determine EVs concentration and size distribution using Nanosight.

### Statistical Analysis

Statistical significance was determined using either Mann-Whitney test or one-way ANOVA. Each experiment was repeated at least three times to determine the biological significance. Data are shown as mean ± SEM and considered statistically significant at *p* < 0.05. GraphPad Prism software (version 7 or 8; GraphPad Software Inc.) was used for analysis.

## Results

### Alcohol Increases Rab GTPases in Primary Human Hepatocytes and in ALD Patients

**FIGURE 1:**
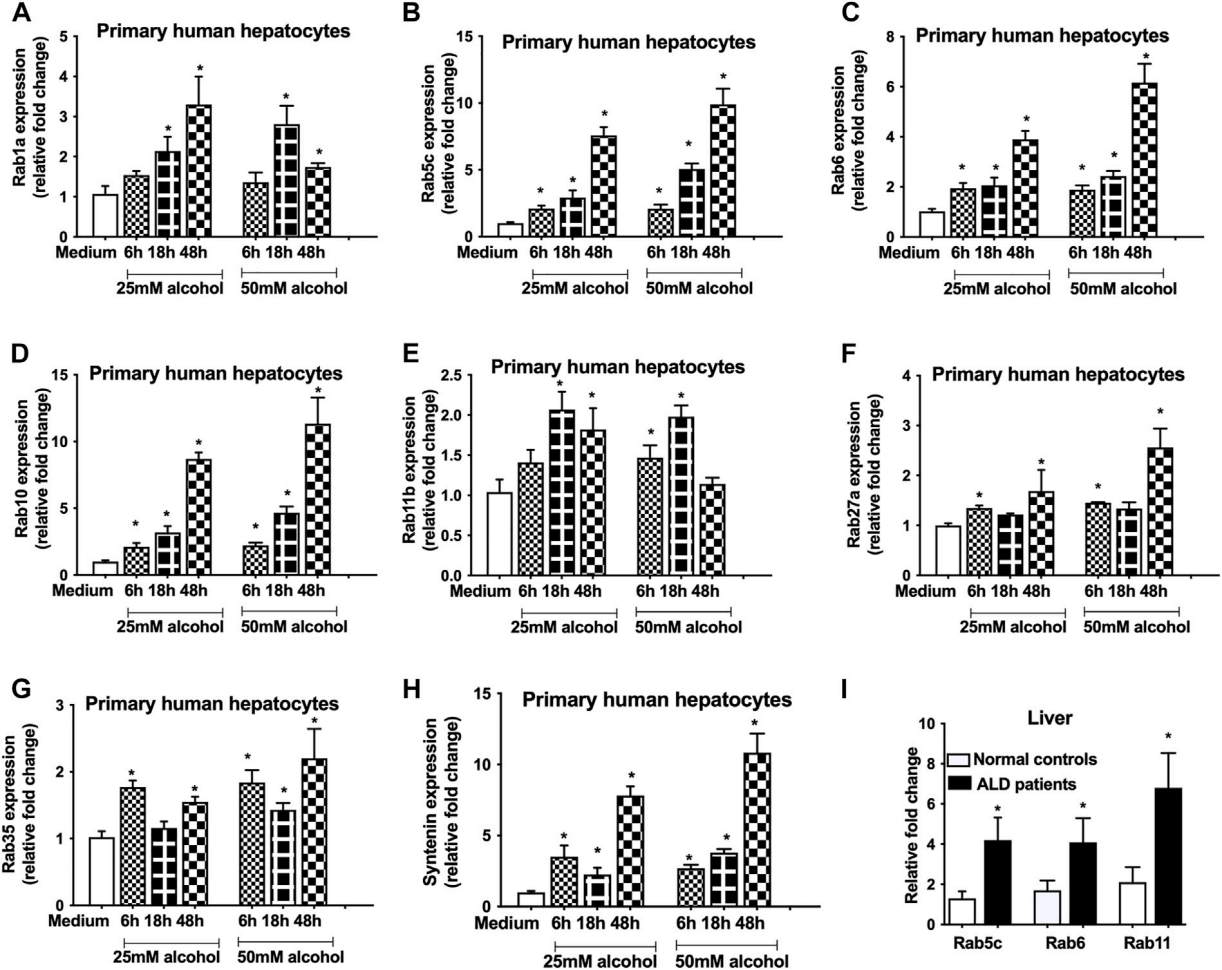
Alcohol induces the expression of Rabs in primary human hepatocytes and in ALD patients.Primary human hepatocytes were cultured and treated with different dosage of alcohol (25 and 50 mM) for indicated times. The expression of Rab1a **(A)**, Rab5c **(B)**, Rab6 **(C)**, Rab10 **(D)**, Rab11b **(E)**, Rab27a **(F)**, Rab35 **(G)** and syntenin **(H)** was determined by qPCR from the total RNA isolated from the cells. Results are representative of three independent experiments. Total RNA was extracted from the livers of normal controls and ALD patients (n = 9) and expression of Rab5c, Rab6 and Rab11 was evaluated by qPCR **(I)**. 18s was used to normalize the cq values. Data represent mean ± SEM. Mann-Whitney test was employed for statistical analysis. *indicates *p* < 0.05 versus control cells **(A-H)** or normal controls **(I)**.

Previously, we showed that alcohol increases exosome release from human hepatocytes (Momen-Heravi et al., 2015a) and exosome numbers were increased in the circulation in alcoholic hepatitis patients (Momen-Heravi et al., 2015b) and in a mouse model of ALD (Momen-Heravi et al., 2015b). However, the mechanism by which alcohol regulates exosome secretion is not known. In this study, we carried out a comprehensive approach to determine the effect of alcohol on genes involved in exosome biogenesis and secretory pathways using human hepatocytes. We found that alcohol increases the transcription of various Rab GTPases involved in exosome biogenesis and secretory pathways. A time dependent increase in Rab1a (18–24 h, Figure 1A), Rab5c (6–48 h, Figure 1B), Rab6 (6–48h, Figure 1C) and Rab10 (6–48 h, Figure 1D), which are the regulators of membrane trafficking and exosome formation, were found in primary human hepatocytes after different doses of alcohol treatment (25 and 50 mM). Rab11, Rab27 and Rab35 have been shown to play a significant direct role in exosome biogenesis and secretion (Blanc and Vidal, 2018) and we found significant increases in the mRNA levels of Rab11b (6–48 h, Figure 1E), Rab27a (6 h, and 48 h, Figure 1F), and Rab35 (6–48 h, Figure 1G) after alcohol treatment. Syntenin, a syndecan binding protein, plays diverse roles in exosome biogenesis (Baietti et al., 2012; Kashyap et al., 2021) and our results indicated a significant induction in its mRNA expression after alcohol treatment (6–48 h, Figure 1H). The kinetics of Rabs tested and syntenin upregulation were similar between 25 and 50 mM of alcohol treatment and highest induction in most of these genes was observed 48 h after alcohol treatment (Figures 1A–H). Next, we determined the expression of some of these Rabs in patients with ALD and found significant increases in Rab5c, Rab6 and Rab11 in ALD patients compared to controls (Figure 1I).

### Alcohol Induces the Expression of Soluble N-Ethylmaleimide Sensitive Factor Attachment Protein Receptors Proteins

**FIGURE 2:**
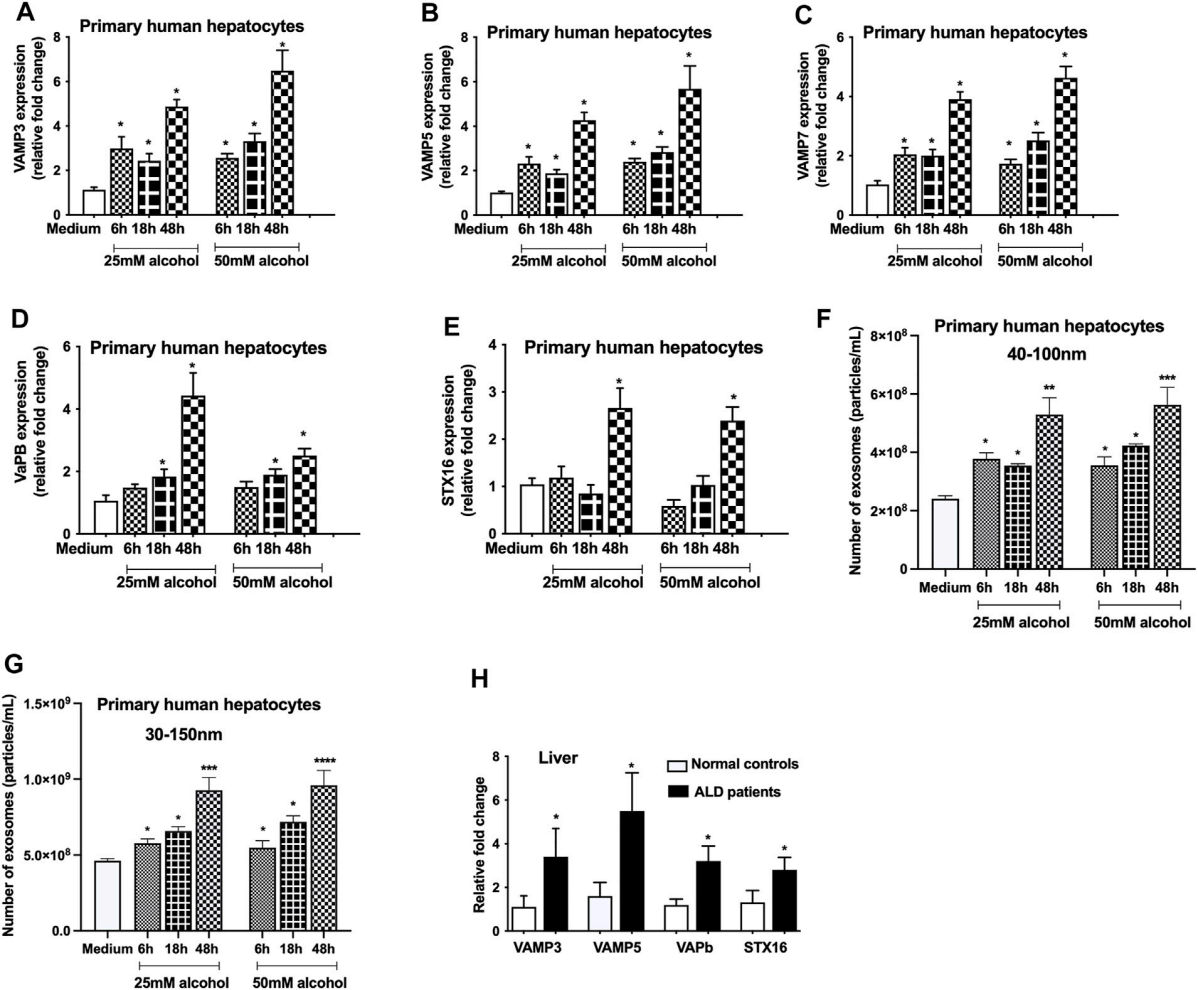
Alcohol increases the expression of VAMPs and syntaxins. Primary human hepatocytes were cultured and treated with different dosage of alcohol (25 and 50 mM) for indicated times and VAMP3 **(A)**, VAMP5 **(B)**, VAMP7 **(C)**, VAPb **(D)**, and STX16 **(E)** expression was assessed by qPCR from the total RNA isolated from the cells. Total number of particles was measured from the cell free supernatant after alcohol treatment (25 and 50 mM) for indicated times using Nanosight **(F and G)**. Data is presented as particles/ml. Number of particles from 40–100 nm and 30–150 nm are represented in F and G, respectively. Total RNA extracted from the livers of normal controls and ALD patients (n = 9) was used to determine the expression of VAMP3, VAMP5, VAPb and STX16 by qPCR **(H)**. 18s was used to normalize the cq values. Data represent mean ± SEM. Mann-Whitney test or one-way ANOVA was used for statistical analysis. *, **, ***,**** indicates *p* < 0.05, *p* < 0.005, *p* < 0.0005, *p* < 0.0001 compared to control cells **(A-G)** or normal controls **(H)**.

The vesicle-associated membrane proteins (VAMPs) belong to v-SNARE family protein, and reside in various post-Golgi vesicular compartments, and mediate vesicle fusion with the plasma membrane (Jahn and Scheller, 2006). To determine the effect of alcohol on VAMPs, we checked the levels of VAMP 3, 5 and 7 and found that alcohol significantly increases the VAMP3 (6–48 h, Figure 2A), VAMP5 (6–48 h, Figure 2B) and VAMP7 (6–48 h, Figure 2C) mRNA transcripts. VAPb, a VAMP associated protein (Chen et al., 2010) was also found to be induced after alcohol treatment (18 and 48 h, Figure 2D). Syntaxin 16 (STX16), belongs to t-SNARE family protein, is required for the accumulation of recycling endosomes (Amessou et al., 2007), and we found increase in STX16 mRNA transcripts only 48 h after alcohol treatment (Figure 2E). Since we found increased expression of Rabs, syntenin and SNAREs, we next determined the EV number and found a time dependent increase in particles ranging from 40 to 100 nm after different doses (25 and 50 mM) of alcohol treatment (6–48 h, Figure 2F). Given that size distribution of particles has been defined as exosomes in the 30–150 nm range by some studies (Tschuschke et al., 2020; Babaei and Rezaie, 2021), we also analyzed our data by measuring the particles from 30 to 150 nm. We found no significant differences in quantifying the particles ranging from 40 to 100 nm and 30–150 nm as shown Figure 2F,G, respectively. It was observed that maximum induction in VAMPs, VAPb, STX16 and exosome number occurred 48 h after alcohol treatment (Figures 2A–G). Significant increases in VAMP3, VAMP5, VAPb, and STX16 were also observed in ALD patients compared to controls (Figure 2H).

### Alcohol Regulates miRNAs Expression at the Transcriptional Level

**FIGURE 3:**
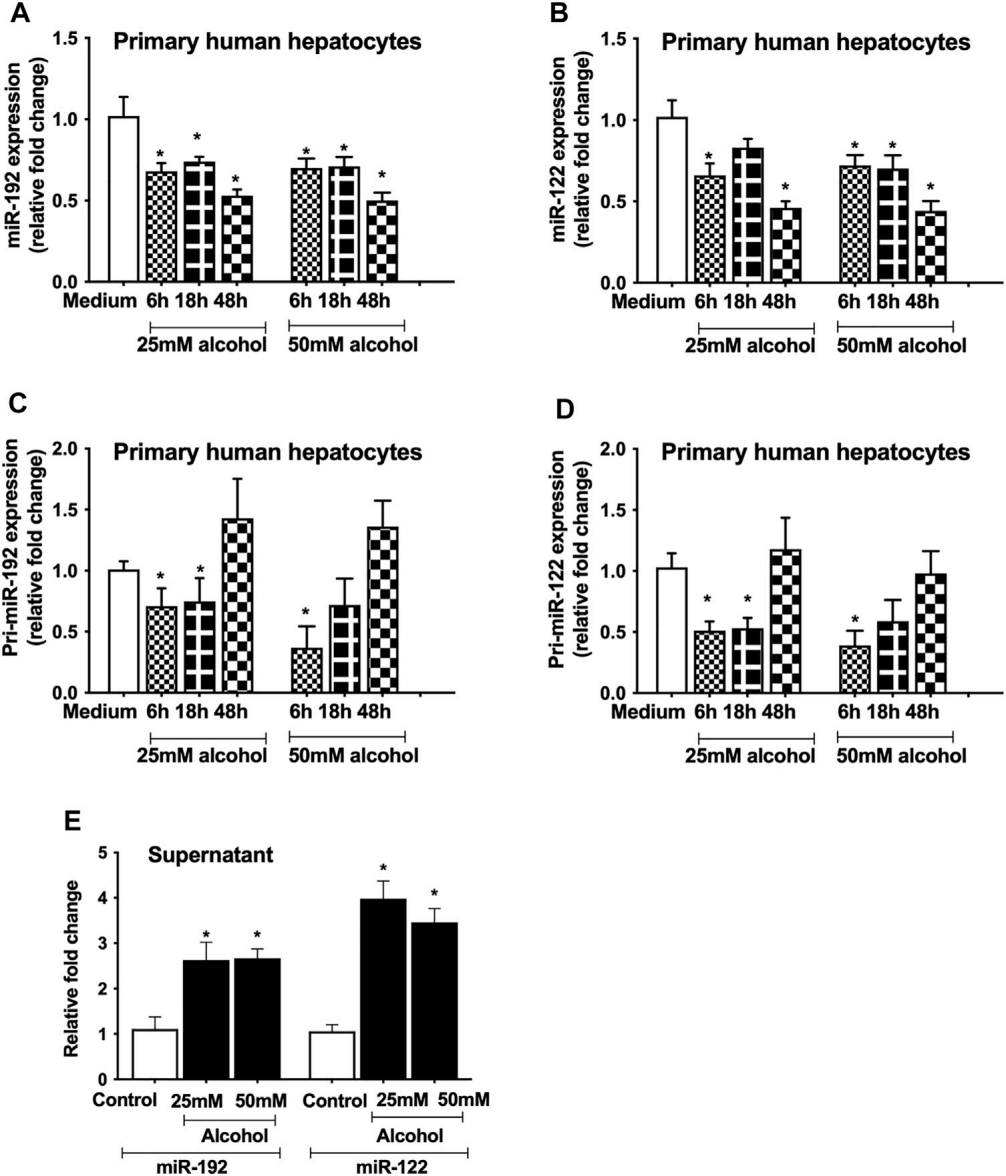
Alcohol regulates miRNA expression at the transcriptional level. Primary human hepatocytes were cultured and treated with different dosage of alcohol (25 and 50 mM) for indicated times. The levels of mature form of miR-192 **(A)** and miR-122 **(B)** were measured by qPCR and RNU48 was used as a normalization control. The primary transcripts of miR-192 **(C)** and miR-122 **(D)** were quantified by qPCR and GAPDH was used as a normalization control. Total RNA was extracted from the cell-free supernatants of primary hepatocytes and miR-192 and miR-122 were evaluated 18 h after alcohol treatment by qPCR and spiked synthetic Cel-miR-39 was used as a normalization control **(E)**. Data represent mean ± SEM. Mann-Whitney test was employed for statistical analysis. *indicates *p* < 0.05 versus control cells.

Since miR-122 and miR-192 are highly expressed in hepatocytes (Momen-Heravi et al., 2015a), we evaluated the effect of alcohol on these miRNAs. A significant decrease in miR-192 (6–48 h, Figure 3A) and miR-122 (6–48 h, Figure 3B) was found in hepatocytes treated with either 25 mM or 50 mM alcohol. To determine the effect of alcohol on the transcriptional levels of these miRNAs, we checked the primary miRNA expression and found a decrease in pri-miR-192 (6 and 18 h, Figure 3C) and pri-miR-122 transcripts (6–18 h, Figure 3D). Previously, we reported increase in miR-192 and miR-122 levels in the exosomes derived from the serum of alcoholic hepatitis patients (Momen-Heravi et al., 2015b) and in a mouse model of ALD (Bala et al., 2012) therefore, we sought to evaluate their levels in the cell-free supernatant. Our results indicated a significant increase in miR-192 and miR-122 levels in the supernatant of primary hepatocytes 18 h after alcohol treatment (25 and 50 mM) (Figure 3E).

### miRNA-192 Regulates the Exosome Secretion *via* Targeting Rabs and Syntaxins

Our bioinformatic analysis revealed that Rab27a, Rab35, STX7 and STX16 are predicted targets of miR-192 (www.microrna.org) (Figure 4A). Since these genes play critical role in exosome secretion (Hsu et al., 2010; Ostrowski et al., 2010), therefore, to determine the causal effect of alcohol-induced miR-192 downregulation on exosome secretion, we carried out simulation experiments using human hepatoma Huh.7.5 cells. First, we determined the optimal time for the induction of these genes in Huh 7.5 cells and found significant increase in Rab27a (6–36 h, Figure 4B), Rab35 (6 and 18 h, Figure 4C), STX7 (18 h, Figure 4D), and STX16 (6–48 h, Figure 4E) mRNA transcripts after alcohol treatment (50 mM). A time dependent increase in exosome (40–100 nm in Figures 4F, 30–150 nm in Figure 2G) secretion (18–48 h) was found, and a maximum increase in exosomes was observed 48 h after alcohol treatment.

**FIGURE 4:**
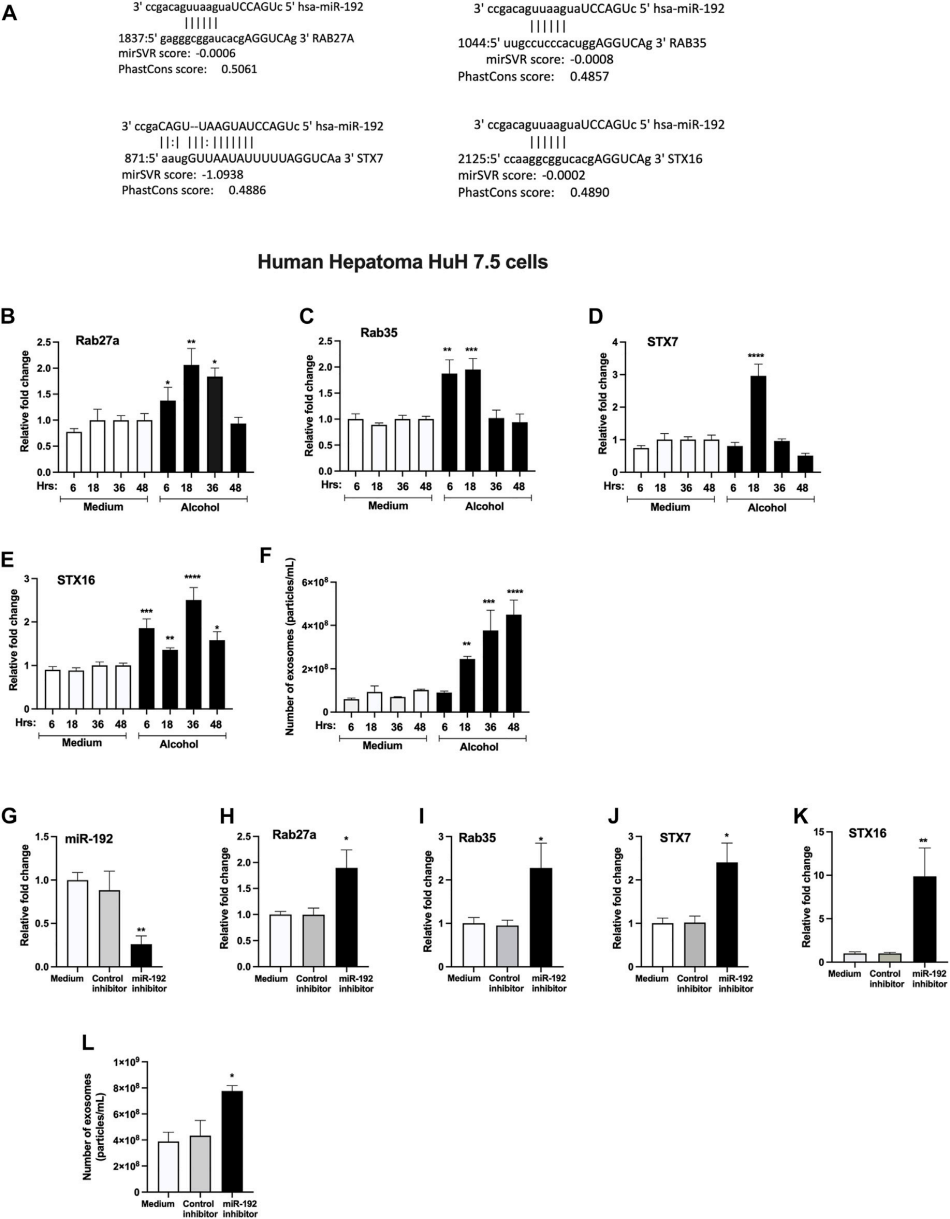
miRNA-192 regulates exosome secretion *via* targeting Rabs and syntaxins.miR-192 putative binding sites at the 3’ UTR of Rab27a, Rab35, STX7 and STX16 genes **(A)**. Huh 7.5 cells were treated or not with 50 mM alcohol for indicated times and expression of Rab27a **(B)**, Rab35 **(C)**, STX7 **(D)**, and STX16 **(E)** was quantified from RNA isolated from cells using qPCR. Total number of particles was measured from the cell free supernatant after 50 mM of alcohol treatment for indicated times using Nanosight and presented as particles/ml **(F)**. Huh 7.5 cells were transfected either with negative control-miRNA or miR-192 inhibitor as described in the methods. miR-192 expression was measured from the cells using qPCR and RNU48 was used as a normalization control **(G)**. The expression of Rab27a **(H)**, Rab35 **(I)**, STX7 **(J)**, and STX16 **(K)** was determined using qPCR. Total number of particles was evaluated from the cell free supernatant after miR-192 inhibition using Nanosight and presented as particles/ml **(L)**. 18s was used to normalize the cq values. Data represent mean ± SEM. One-way ANOVA or Mann-Whitney test was used for statistical analysis. *, **, ***, ****indicates *p* < 0.05, *p* < 0.005, *p* < 0.0005, *p* < 0.0001 compared to control cells.

Transfection of a miR-192 inhibitor in hepatocytes caused a significant decrease in miR-192 levels compared to control inhibitor cells (Figure 4G). miR-192 inhibition resulted in a significant increase in Rab27a (Figure 4H), Rab35 (Figure 4I), STX7 (Figure 4J), and STX16 (Figure 4K). Furthermore, an approximately 1.8-fold induction in exosome secretion was detected in cells transfected with miR-192 inhibitor compared to control inhibitor treated cells (Figure 4L).

## Discussion

**FIGURE 5:**
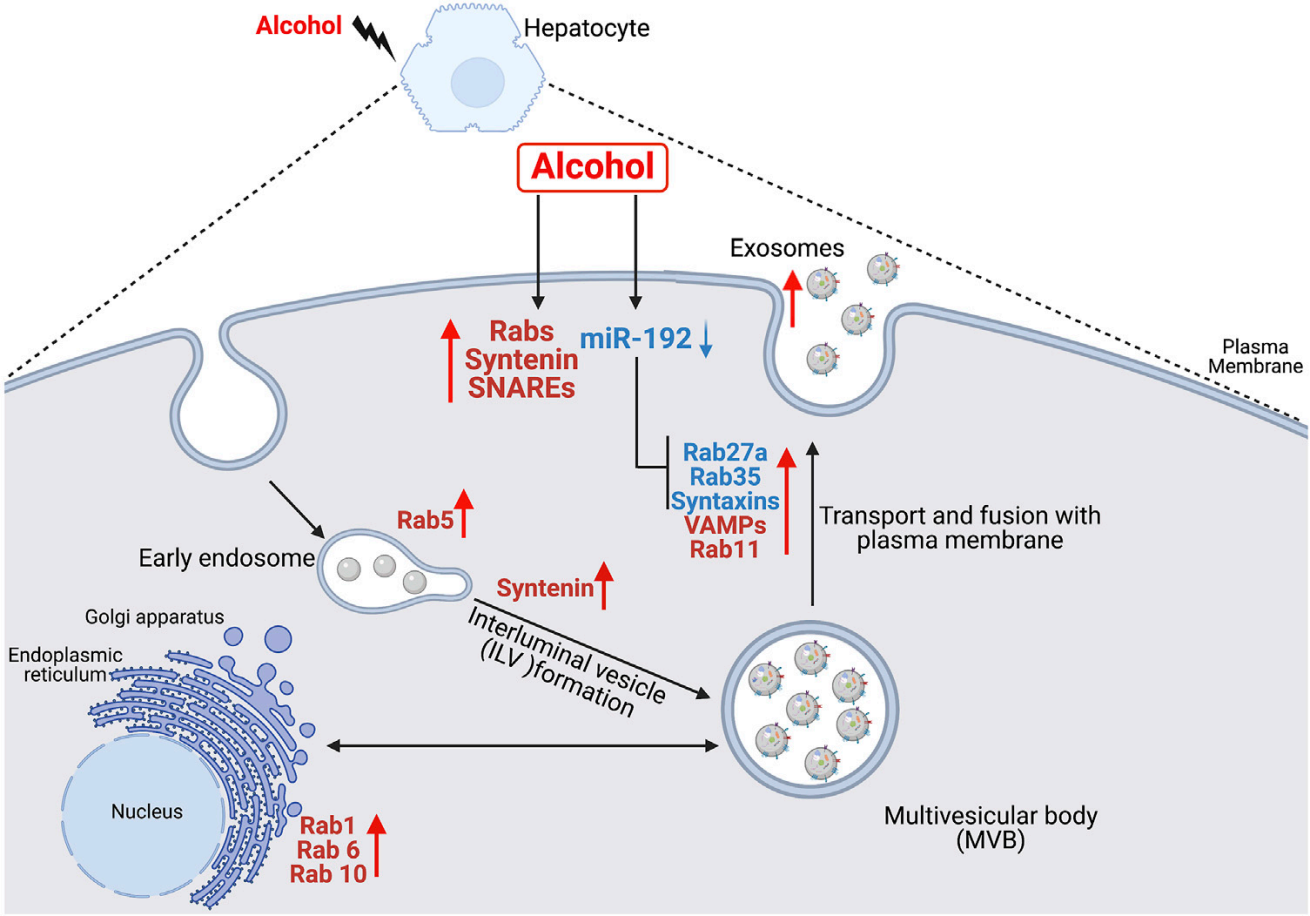
Schematic representation of the effect of alcohol on exosome biogenesis and secretion in human hepatocytes. Alcohol modulates the expression of various Rabs, syntenin, VAMPs and syntaxins directly or partly *via* miR-192 in hepatocytes. Alcohol-induced decrease in miR-192 results in increase in miR-192 target genes (Rab27a, Rab35, syntaxin7 and syntaxin16) and a concurrent induction in exosome secretion.

Various studies have shown increased secretion of exosomes during cellular stress (Momen-Heravi et al., 2015a; Momen-Heravi et al., 2015b; Hessvik and Llorente, 2018; Babuta et al., 2019; Babuta and Szabo, 2021). Majority of alcohol is metabolized in hepatocytes and alcohol metabolites induce oxidative stress (Momen-Heravi et al., 2015a). Continuous use of alcohol results in hepatocyte damage and subsequently to alcohol-associated liver disease (ALD) (Momen-Heravi et al., 2015b). Previously, we showed that alcohol increases exosome production in human hepatocytes (Momen-Heravi et al., 2015a), in alcoholic hepatitis patients (Momen-Heravi et al., 2015b) and in mouse models of ALD (Momen-Heravi et al., 2015a; Momen-Heravi et al., 2015b). In this study, we investigated the effect of alcohol on genes involved in the formation of exosomes (biogenesis, transport and fusion to the plasma membrane). Here, we report three major findings, first, alcohol induces the transcription of various Rabs, and v- and t- SNAREs in human hepatocytes and in ALD patients. Second, alcohol downregulates miR-192 and promotes exosome secretion. Third, miR-192 regulates exosome secretion in human hepatocytes *via* targeting Rab27a, Rab35 and syntaxins. To our best knowledge this is the first study demonstrating the effect of alcohol on exosome biogenesis and secretory pathways directly and partly *via* miR-192 and a link between miR-192 and exosome secretion. These findings are illustrated in Figure 5.

Syntenin, a syndecan binding protein, plays various roles in exosome biogenesis (Ghossoub et al., 2014; Hessvik and Llorente, 2018; Kashyap et al., 2021). It stimulates exosome production *via* its interaction with syndecan-ALIX-ARF6 complex and other factors (Ghossoub et al., 2014). A recent study signifies that syntenin not only involves in the endosomal budding of cargo (biogenesis) and exosome production, but also regulates the uptake of exosomes (Kashyap et al., 2021). Our results suggest an induction in syntenin in alcohol treated hepatocytes suggesting a potential role of syntenin in exosome production in ALD, this observation deserves further investigation.

Rab GTPases (Rab proteins) are well recognized for their role in vesicular trafficking and exosome formation (Blanc and Vidal, 2018). These proteins regulate a distinct intracellular transport step and are found in exosomes (Blanc and Vidal, 2018). Various Rabs such as Rab11, Rab27 and Rab35 are shown to play essential direct role/s in exosome biogenesis and secretion (Ostrowski et al., 2010; Ramel et al., 2013). Rab11 regulates transferrin receptor secretion *via* the exosome pathway (Ramel et al., 2013) whereas Rab35 promotes exosome release by interacting with its effector protein (Hsu et al., 2010). Rab27 is involved in MVE docking to the plasma membrane (Ostrowski et al., 2010). Both isoforms of Rab27, Rab27a and Rab27b are shown to play a common and different role in exosome secretion by promoting the targeting of MVEs to the cell periphery and docking to the plasma membrane (Ostrowski et al., 2010). We previously showed increase in Rab27b after alcohol treatment (Momen-Heravi et al., 2015a), and in this study, we found Rab27a, and not Rab27b, is a miR-192 predicted target gene. Therefore, we focused on Rab27a.

Other Rabs (Rab1, 5,6 and 10) that we found upregulated after alcohol treatment, have been indicated to play various roles in vesicular transport. Rab1 is known to mediate dynamic membrane trafficking between endoplasmic reticulum and Golgi (Yang et al., 2016) and Rab5 has been proposed to be a master regulator of endosome biogenesis and trafficking (Nagano et al., 2019). Rab6 is a Golgi associated Rab that modulates the constitutive secretory pathway (Patwardhan et al., 2017). Rab10 is present in endoplasmic reticulum and common endosomes, and it was reported to mediate transport from basolateral sorting endosomes to common endosomes (Babbey et al., 2006)**.** Consistent with their role in exosome secretion, we found increase in these Rabs within alcohol treated hepatocytes. Considering the roles of these Rabs in vesicular transport, it is likely that alcohol in general modulates the vesicle trafficking between organelles.

SNARE family proteins play critical roles in all fusion reactions of the secretory pathway and are classified either as vesicular (v-SNARE) and target (t-SNARE) membrane based on their primary subcellular localization (Jahn and Scheller, 2006). VAMPs belong to v-SNARE proteins where as syntaxins belong to t-SNARE proteins, and both are involved in various stages of membrane fusion pathway (Jahn and Scheller, 2006). Our results indicated that alcohol upregulates the expression of VAMP3, VAMP5 and VAMP7 and synatxin16 in hepatocytes and increased levels of these SNAREs were also found in ALD patients. VAMP3, VAMP7 and syntaxin16 were shown to mediate fusion between MVBs with autophagosomes and autolysosome formation (Miller and Shindell, 1998; Tang, 2019), and we recently described a link between miR-155 and lysosome dysfunction and exosome production (Babuta et al., 2019). VAMP7 was also demonstrated to participate in the fusion between MVBs with the plasma membrane to release exosomes (Fader et al., 2009). It is important to note that these proteins (Rabs, syntenin, and SNAREs) are not only involve in the exosome trafficking but also in the transport of other vesicles and in other processes, suggesting that alcohol overall affects the vesicle trafficking and, thereby, has a vast impact on cellular homeostasis.

miR-192 regulates various processes like oxidative stress, cellular proliferation, and inflammatory responses (Fuschi et al., 2017; Ren et al., 2021). miR-192 is the second most expressed miRNA in hepatocytes and is shown to play diverse roles in liver diseases including Non-alcoholic fatty liver disease (NAFLD), drug-induced liver injury and hepatocellular carcinoma (Ren et al., 2021). In a rat model of NAFLD, a decrease in hepatic miR-192 expression has been reported and is shown to involve in the regulation of lipid metabolism and inflammation (Liu et al., 2017). In the present study, we report that alcohol downregulates miR-192 and miR-122 expression in hepatocytes *via* affecting its transcription. Though we found decrease in pri-miRs at 6 and 18 h of alcohol treatment, the mature miRs remained decreased even 48 h after alcohol treatment suggesting alcohol affects other factors that modulate miRs expression at the post-transcriptional level. For instance, mature miR-192 expression is shown to be regulated (inhibited) at the post-transcriptional level by various factors such as p53, TGFβ etc. (Ren et al., 2021) and increase in p53 and TGFβ was reported in alcohol-associated liver disease (Zhou et al., 2019). Since miR-122 locus is under circadian control, it was shown that levels of pri and pre-miR-122 oscillates during the day whereas mature miR-122 remains constant due to its high stability (Gatfield et al., 2009).

While we found decrease in mature miRs in hepatocytes, an induction in these miRs was observed 18 h after alcohol treatment in the cell-free supernatant. Previously, we found that alcohol causes increase in miR-192 and miR-122 levels into exosomes and is an active process (Momen-Heravi et al., 2015a). So, it is likely that majority of miR-192 and miR-122 in the supernatant are associated with exosomes. There are several factors such as RNA binding proteins (Ago2, hnRNPs and others), cellular stress, membrane proteins (such as neural sphingomyelinase 2) and the miRNA induced silencing complex (miRISC)-related pathway that affect the sorting of miRNA/cargoes into exosomes (Zhang et al., 2015; Fabbiano et al., 2020; Groot and Lee, 2020; Babuta and Szabo, 2021). The main components of miRISC include miRNA, miRNA-repressible mRNA, GW182, and Ago2 (Zhang et al., 2015; Babuta and Szabo, 2021), and we previously demonstrated that alcohol induces the expression of GW182 (Bukong et al., 2013). It is possible that alcohol is modulating the expression of these proteins to induce miRs sorting into exosomes. This observation needs to be investigated further.

While we found a similar expression change for both miR-192 and miR-122, miR-192 was selected for mechanistic studies because it exhibits binding sites for several genes in the exosome biogenesis pathway. Our bioinformatic target scan analysis revealed that Rab27a, Rab35, STX7 and STX16 are the predicted miR-192 genes (www.microrna.org) and our mechanistic studies provided the evidence that miR-192 regulates these genes and modulates the exosome secretion in hepatocytes. Various studies have described the enrichment of exosomes with cholesterol, sphingomyelin, and ceramide etc. and suggested that exosome composition resembles that of plasma membrane rafts (Egea-Jimenez and Zimmermann, 2020). Since miR-192 is shown to regulate lipid metabolism (Liu et al., 2017), therefore, it is possible that it has a larger role in exosome secretion. It is highly likely that miR-192 regulates exosome secretion *via* having its effect on multiple genes involved in the biogenesis and secretory pathways and lipid metabolism. Further studies will aid in deciphering the precise role of miR-192 in exosome secretion. Increased miR-192 levels have been reported in biofluids and exosomal miR-192 is suggested as a potential biomarker of the disease progression (Ren et al., 2021). Previously, we demonstrated increase in exosomal miR-192 in alcoholic patients and in a mouse model of ALD (Momen-Heravi et al., 2015b). In the present study, we showed increased levels of miR-192 in the supernatant of alcohol treated hepatocytes. Since miR-192 is enriched in hepatocytes it is likely that hepatocytes are the source of alcohol-induced increase in miR-192 in the exosomes. Hepatocytes are major type of hepatic cells, occupy about 80% of the total liver volume and is shown to communicate with other hepatocytes and other liver cells *via* exosomes (Sung et al., 2018). Exosomes from palmitic acid-treated hepatocytes resulted in the induction of hepatic stellate cell (HSC) activation and further analysis revealed that exosomal miR-192 caused increase in the expression of profibrotic gene markers in HSC (Lee et al., 2017). We previously showed that exosomes isolated from alcohol treated hepatocyte can reprogram monocytes *via* miR-122, inducing sensitization to LPS and increased levels of pro-inflammatory cytokines (Momen-Heravi et al., 2015a). The stem cell-derived exosomes have been shown to promote angiogenesis both *in vitro* and *in vivo* experimental conditions and demonstrates beneficial effects on ischemic diseases (Babaei and Rezaie, 2021).

In conclusion, our study reveals that alcohol promotes exosome secretion *via* affecting genes involved in exosome biogenesis, transport, and secretion. Further, our results suggest that miR-192 as one of the regulators of exosome secretion in alcohol-induced cellular stress.

## Data Availability

The raw data supporting the conclusions of this article will be made available by the authors, without undue reservation.
